# Design, fabrication and characterization of Computer Generated Holograms for anti-counterfeiting applications using OAM beams as light decoders

**DOI:** 10.1038/s41598-017-18147-7

**Published:** 2017-12-21

**Authors:** Gianluca Ruffato, Roberto Rossi, Michele Massari, Erfan Mafakheri, Pietro Capaldo, Filippo Romanato

**Affiliations:** 1 Department of Physics and Astronomy ‘G. Galilei’, University of Padova, via Marzolo 8, 35131 Padova, Italy; 2 LaNN, Laboratory for Nanofabrication of Nanodevices, EcamRicert, Corso Stati Uniti 4, 35127 Padova, Italy; 3 CNR-INFM TASC IOM National Laboratory, S.S. 14 Km 163.5, 34012 Trieste, Basovizza Italy

## Abstract

In this paper, we present the design, fabrication and optical characterization of computer-generated holograms (CGH) encoding information for light beams carrying orbital angular momentum (OAM). Through the use of a numerical code, based on an iterative Fourier transform algorithm, a phase-only diffractive optical element (PO-DOE) specifically designed for OAM illumination has been computed, fabricated and tested. In order to shape the incident beam into a helicoidal phase profile and generate light carrying phase singularities, a method based on transmission through high-order spiral phase plates (SPPs) has been used. The phase pattern of the designed holographic DOEs has been fabricated using high-resolution Electron-Beam Lithography (EBL) over glass substrates coated with a positive photoresist layer (polymethylmethacrylate). To the best of our knowledge, the present study is the first attempt, in a comprehensive work, to design, fabricate and characterize computer-generated holograms encoding information for structured light carrying OAM and phase singularities. These optical devices appear promising as high-security optical elements for anti-counterfeiting applications.

## Introduction

The increasing need for ID security and brand protection is driving global adoption of sophisticated technologies to provide considerable and effective barriers to counterfeit through the use of security holograms^1^. In response to this demand, holographic industries devote a big afford to enhance intrinsic material properties and fabrication complexity^2^. Likewise, fraud and counterfeiting techniques evolve and the majority of conventional security features are compromised. In order to prevent forgery attempts, the holographic patterns are constantly implemented by increasing the security level, reaching sometimes a degree of sophistication that overwhelms the original intent of fraud prevention. Even if today the development of micro-ad nano-scale fabrication techniques can produce optical and physical features virtually impossible to counterfeit, on the other hand it is important to provide easy detection methods to the examiners^3^.

Concurrently, optical security and encryption marked significant progress and advancement since the double random phase encoding (DRPE) method was published^4^. In the last decades, many variations of this technique have been reported^5,6^ and more solutions have been presented and outlined in order to improve the optical security of information^7,8^. An advantage of optical encryption has been its ability to use multiple degrees of freedom to generate complex multi-dimensional security keys including wavelength, polarization, 3D coordinates and complex amplitude, both in classical and single-photon regimes^9^.

With the intent of engineering novel easy-to-read security optical elements, in this work we focus on the degree of freedom represented by the decoding illumination and we design a new type of diffractive optical elements (DOEs) which correctly decode visual information only when illuminated with light owning specific spatial distributions of intensity and phase. As numerical analysis and preliminary experimental results suggested^10^, it is possible to consider these high-security optical elements as a further advancement in anti-fraud technologies.

Traditionally, a hologram is referred to as a physically-recorded interference pattern between a coherent reference beam and the wave scattered by an object^11^. The presence of the actual physical object is nowadays not necessary since the mathematical relations between object and image fields can be implemented numerically. In computer-generated holograms (CGHs), in fact, the hologram pattern is numerically designed and optimized, in terms of signal to noise ratio (SNR) and diffraction efficiency *η* of the reconstructed image^12^. The physical process that allows the reconstruction of the image in far-field through a diffraction mechanism occurring in the holographic pattern is mathematically expressed by the Fresnel-Kirchhoff diffraction equation^13^. In the paraxial approximation, this relation provides the diffraction field *O*(*x, y, z*) generated at a distance *z* from a diffractive optical element, and it is given by:

1 $$O(x,y,z)=\frac{{e}^{ikz}}{i\lambda z}\int {\int }_{S}{U}^{i}(x\text{'},y\text{'})G(x\text{'},y\text{'}){e}^{ik\frac{{(x-x\text{'})}^{2}+{(y-y\text{'})}^{2}}{2z}}dx\text{'}dy\text{'}$$

where *U* ^*i*^ (*x*′*, y*′) is the complex field incident on the holographic plane, *G* (*x*′*, y*′) is the DOE transmission function, (*x*′*, y*′) and (*x, y*) are the Cartesian coordinates on the holographic plane and on the image plane respectively, and *k* = *2π / λ* is the incident wave vector, being *λ* the incident wavelength. By developing the two square terms in the exponential contribution inside the integral in eq. (1), we get the following form:

2 $$O(x,y,z)=\frac{{e}^{ikz}}{i\lambda z}{e}^{ik\frac{{x}^{2}+{y}^{2}}{2z}}FT[{A}^{\ast }](\frac{x}{\lambda z},\frac{y}{\lambda z})$$

where *FT* stands for the Fourier Transform operator. Therefore the diffraction field *O*(*x, y, z*) is related to the Fourier Transform of a modified hologram transmission function *A* ^*^, calculated at the spatial frequencies (*x/λz*, *y/λz*). The transmission function *A* ^*^ is defined as:

3 $${A}^{\ast }(x\text{'},y\text{'})={U}^{i}(x\text{'},y\text{'})G(x\text{'},y\text{'})\exp (ik\frac{x{\text{'}}^{2}+y{\text{'}}^{2}}{2z})$$

and results to be the product between the hologram phase function, the incident field on the hologram plane, and the Fresnel phase factor. Previous equations serve as the basis for the computation of Fresnel computer-generated holograms with specific incident illumination.

**Figure 1:**
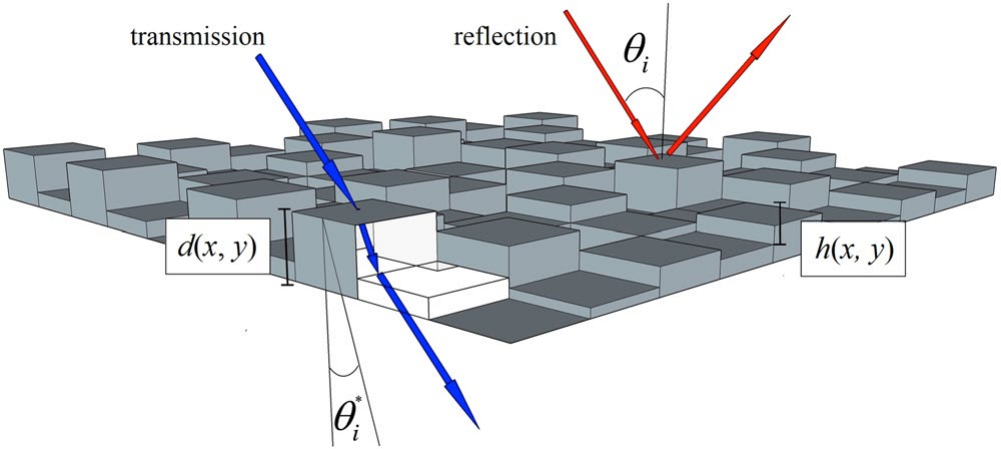
Schematic representation of the light path interacting either in transmission or in reflection with a multilevel phase-only diffractive optical element (PO-DOE).

Diffractive optics can be designed to work either in transmission or in reflection and can be engineered to manipulate either the phase or the amplitude (or both) of the input wave. Due to their higher efficiency, phase-only holograms are far more preferable. The phase control of the holographic pattern is expressed by its phase function *φ*(*x, y*), which is given below for the two different configurations. For transmission holograms, we have:

4 $$\phi (x,y)=\frac{2\pi }{\lambda }\,\cos ({\vartheta }_{i}^{\ast })[n(\lambda )-1]d(x,y)$$

being *ϑ*_i_^*^ the propagation angle inside the hologram medium, *n*(*λ*) the refractive index for the given wavelength, *d*(*x, y*) the local thickness of the hologram at the position (*x, y*). In case of holograms working in reflection, we have instead:

5 $$\phi (x,y)=\frac{4\pi }{\lambda }\,\cos ({\vartheta }_{i})h(x,y)$$

defining *ϑ*_*i*_ as the incident angle of illumination and *h*(*x, y*) as the depth of the hologram pattern at the coordinates (*x, y*). A schematic representation of the light path interacting either in transmission or in reflection with a multilevel phase-only diffractive optical element (PO-DOE) is shown in Fig. 1.

Once the phase pattern of the diffractive optics has been computed, last equations allow the implementation of the holographic nanostructured surface.

After the seminal paper of Allen and co-workers in 1992^14^, it is known that light beams characterized by helicoidal phase-fronts possess a well-defined orbital angular momentum (OAM). Such beams are characterized by a phase term *exp*(*iℓφ*), being *ℓ* the amount of OAM carried by each single photon in units of *ħ*. Since then, light beams carrying OAM have gained increasing attention due to the wide range of uses and different possible applications^15–18^, such as: particle trapping^19^ and tweezing^20^, phase contrast microscopy^21^, STED microscopy^22^, quantum-key distribution^23^ and telecommunications^24^. In the paraxial regime, an OAM beam can be described in terms of Laguerre-Gaussian Modes (LG) characterized by two indices *ℓ* and *p*, the azimuthal and radial index respectively. The azimuthal index *ℓ*, corresponding to the topological charge of the embedded phase singularity, represents the number of intertwined helical wave-fronts. The index *p* represents the number of radial nodes on a plane perpendicular to the direction of propagation and it is related to the distribution of the intensity pattern in *p* + 1 concentric rings around the central dark zone of the phase singularity.

Different techniques have been presented to tailor the orbital angular momentum of a light beam, such as astigmatic mode converters^25^, fork-holograms^26^ and *q*-plates^27^. In this work, we use a method based on transmission through spiral phase plates (SPPs). SPPs are phase optical elements looking like spiral staircases, which are able to shape an incident Gaussian beam into an OAM beam, as shown by Beijersbergen *et al*.^28^. Common SPPs are transparent optical elements whose thickness *h* increases as a function of the azimuthal coordinate according to:

6 $$h(r,\phi )=\ell \frac{\phi }{2\pi }\frac{\lambda }{{n}_{SPP}-{n}_{0}}$$

where *n*_*SPP*_ is the refractive index of the SPP material, *n*_0_ is the refractive index of the surrounding medium, usually air, and *λ* is the impinging wavelength.

**Figure 2:**
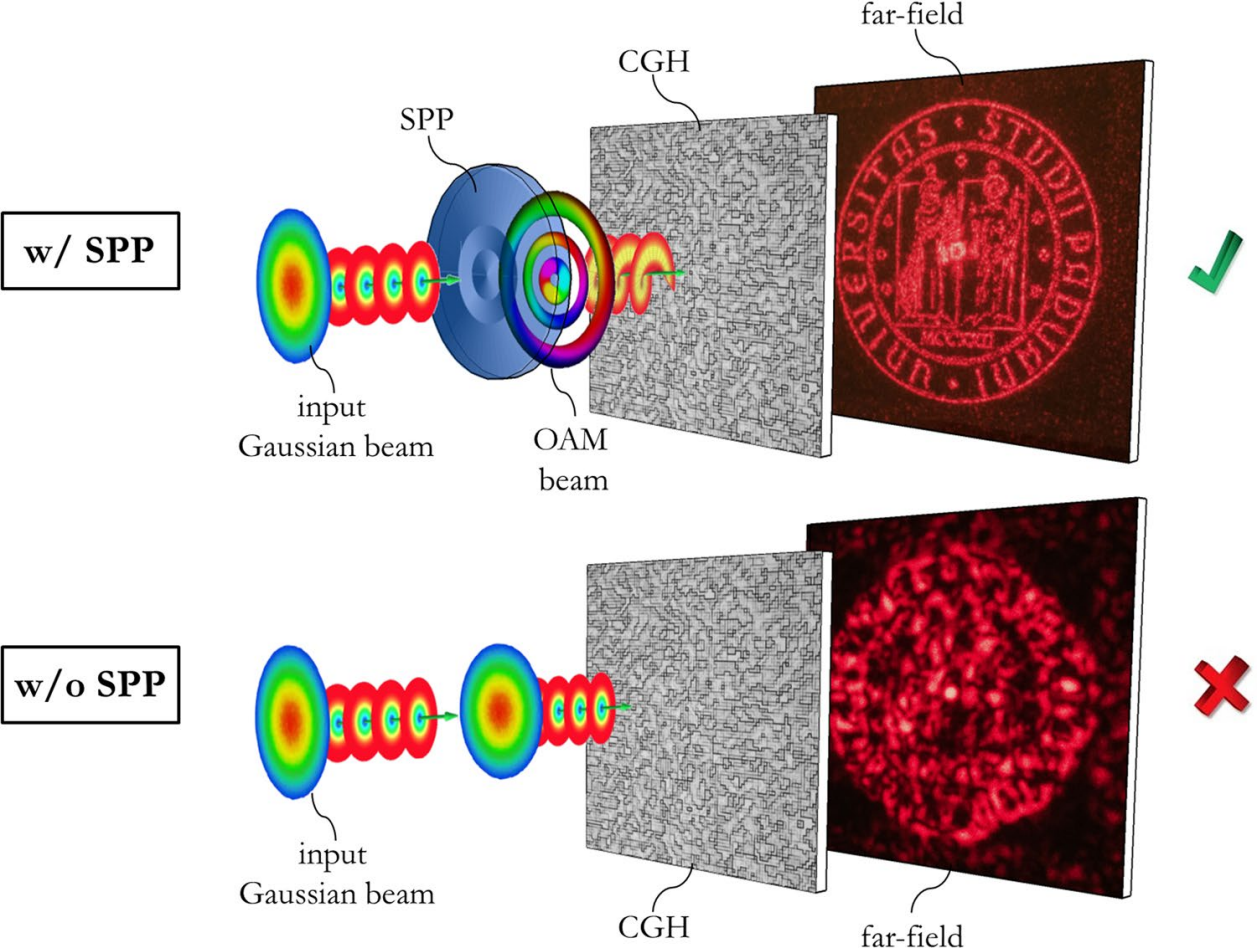
Computer-generated holograms (CGH) working principle. Decoding of the CGH with the correct structured-light generated by the SPP (w/ SPP): the encoded image (University of Padova logo) appears in far-field (at the top). Illumination with standard Gaussian illumination (without (w/o) SPP): the image is not recognizable (at the bottom). The University of Padova logo is © University of Padova and used with permission.

Detailed work has been done in order to optimize both design and fabrication procedures for the generation of high-order OAM beams with non-zero radial index^29^. This is feasible by introducing radial π-discontinuities on the SPP phase pattern Ω_SPP_:

7 $${{\rm{\Omega }}}_{SPP}(r,\phi )=\ell \phi +\frac{\pi }{2}\{1-\mathrm{sgn}[{L}_{p}^{|\ell |}(\frac{2{r}^{2}}{{w}_{0}^{2}})]\}$$

where *L*_*p*_^*|ℓ|*^ is the associated Laguerre polynomial and *w*_0_ the beam waist of the generated LG beam. In our case, the SPP plays the fundamental key-role to generate the light beam decoding the specifically-designed computer-generated hologram, expanding the range of possible application whenever information needs to be stored with increased security and counterfeit prevention. With respect to other optical encryption techniques, a deterministic distribution of intensity and phase is exploited for information encoding into a holographic form. The input light state can be labelled with a set of indices, given arbitrarily, defining the intensity distribution and phase pattern in the selected family of beams. In the specific, we considered beams carrying orbital angular momentum of light and generated with custom spiral phase plates, identified by the OAM content *ℓ* and the radial index *p*. Samples have been fabricated by electron-beam lithography on polymethylmethacrylate (PMMA) resist layer, spun over a glass substrate, in high-resolution mode, providing high-quality phase-only diffractive optics. In addition, since this fabrication technique, however extremely precise, is slow and expensive, we investigated the possibility to replicate the fabricated optics with faster mass-production techniques, such as nanoimprinting^30^, which allows higher throughput and much lower production costs. The optical response has been tested on an optical table, showing a correct reconstruction of the encoded information under illumination with the expected OAM field. Conversely, if the computer-generated hologram is illuminated with a common Gaussian beam, the noise is too high to make the image recognizable (Fig. 2). This study refers to a particular case of computer-generated holography, therefore the reader should consider ‘hologram’ standing for ‘computer-generated hologram’ throughout the text.

## Results

### Holographic design and computation

The realization of a computer-generated hologram can be schematically split into three steps: analysis, implementation and fabrication. The first one consists in the understanding of the physical process governing the formation of the image encoded on the holographic substrate. Therefore, the hologram phase pattern is implemented through the development of a numerical algorithm, which considers both the physics governing the image formation and the restrictions imposed by the selected fabrication process, e.g. limited resolution and spatial mesh. Finally, the designed pattern is realized, with the properly selected fabrication techniques and protocols.

An Iterative Fourier Transform Algorithm (IFTA) represents a proper choice for the design of computer-generated holograms, due to the capability of generating an optimized phase pattern by bouncing back and forth the information between two spaces related by a Fourier transform, i.e. the hologram plane and the image plane. In the specific, the developed code implements a modified version of the Gerchberg-Saxton (GS) algorithm, which since its first publication in 1972^31^ has known many improvements and applications for the calculation of computer-generated holograms^32–34^, with particular attention to phase-only CGH^35–37^, due to their higher efficiency.

**Figure 3:**
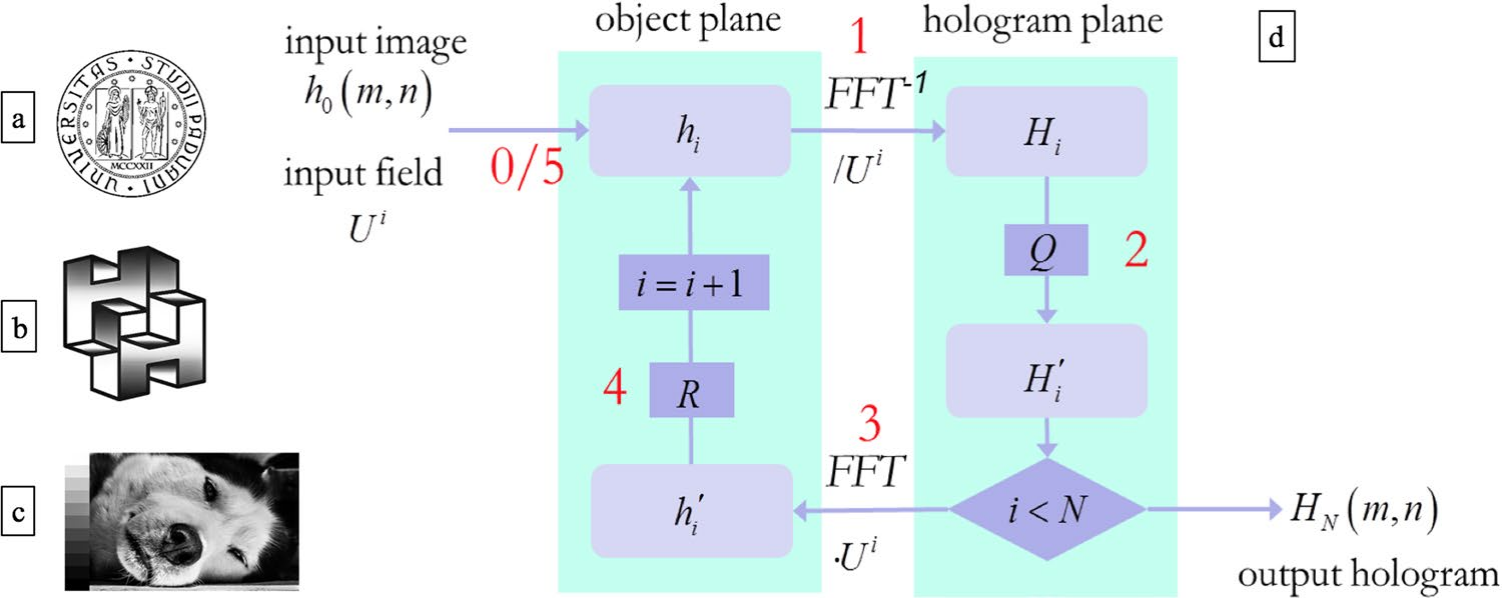
Processed images for the computer-generated holograms. (**a**) UniPD Logo bitmap format with pure black and white pixel, (**b**) two intersecting ‘H’ with a grayscale 8 bit/channel and (**c**) wolf portrait characterized by a 8 bit/channel grayscale with finer details. (**d**) Schematic representation of the iterative Fourier transform algorithm. After the signal input of the *i*th iteration step in the object domain enters the loop, the inverse fast Fourier transform (FFT^−1^) allows the transition to the hologram plane with the hologram function *H*_*i*_ (1), before normalization with respect to the incident field *U* ^*i*^, hence the quantization operator *Q* is applied for both direct partial quantization of phase and amplitude elimination (2). The discretized hologram pattern *H*_*i*_ *′* is multiplied by the incident field *U* ^*i*^ and the fast Fourier transform (FFT) is performed (3), obtaining the corresponding reconstructed field hi′ in the object domain. Then the final new signal *h*_*i+*1_ is obtained with the proper replacement of the output signal amplitude with the desired image amplitude within the signal window (4). The loop is repeated (5) for *N* iterations, until convergence. The University of Padova logo is © University of Padova and used with permission.

In Fig. 3(d), a scheme of the GS algorithm implemented in MATLAB^®^ environment, is shown. The process begins by collecting the experimental amplitude field generated by the selected SPP. The phase pattern, for given indices *p* and *ℓ* of the generating SPP, is assumed to have an azimuthal dependence of the form *ℓφ*, plus radial jumps equal to π in correspondence of the zeros of the associated Laguerre polynomial *L*_*p*_^*ℓ*^, as given by eq. (7). Previous interferometric analyses^38^ on the OAM beams generated by the fabricated SPPs confirm the validity of this assumption. The collected intensity distribution and the corresponding azimuthal phase gradient define the complex input field for the computation of the hologram phase pattern.

**Figure 4:**
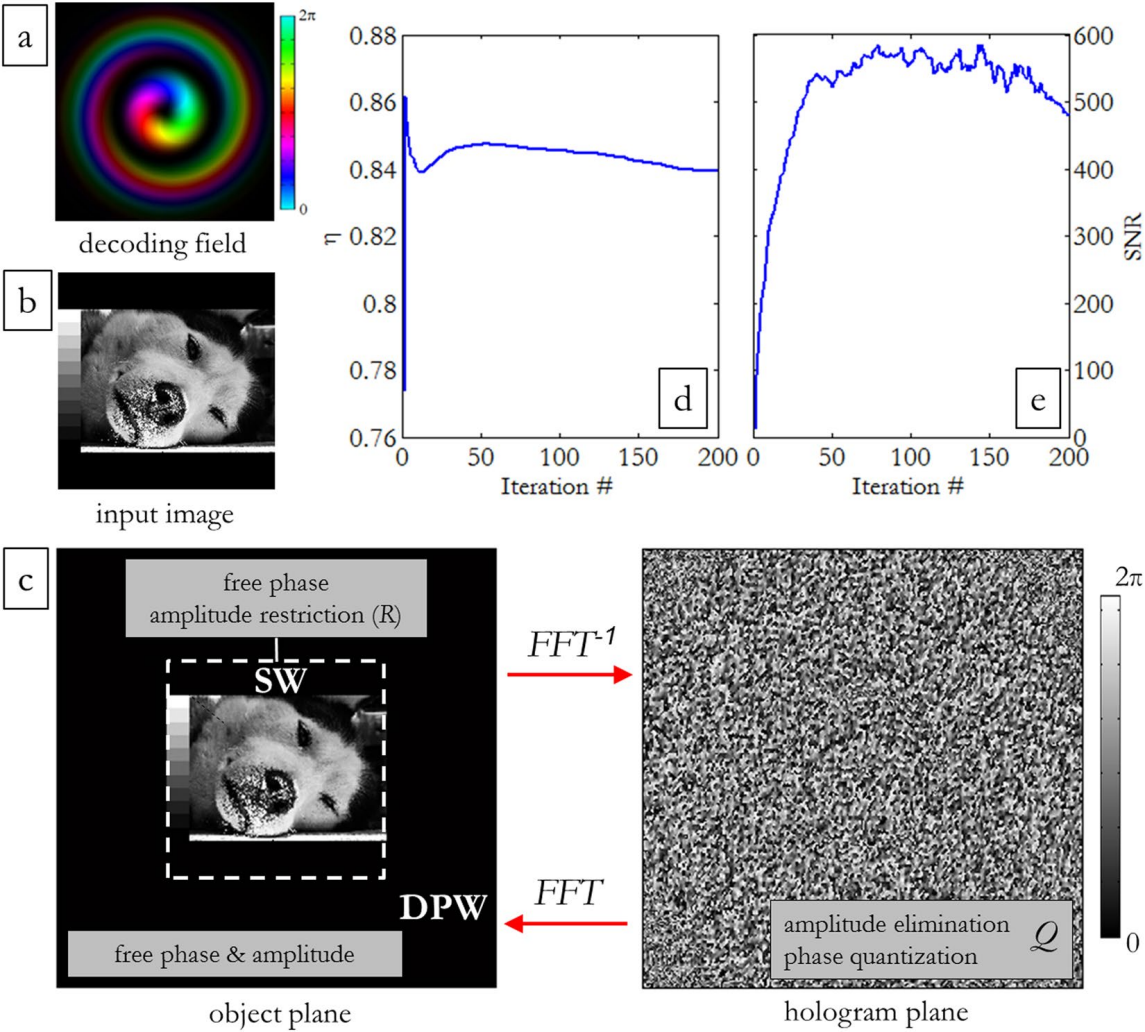
(**a)** Experimental beam generated by SPP with *p* = 1, *ℓ* = 1. Brightness and colours refer to intensity (experimental) and phase (theoretical) respectively. (**b**) Input image entering the optimization loop. (**c**) On the left: diffracted pattern window (DPW) on the object plane, including the signal window (SW) where the image is formed. At each step of the algorithm, a replacement of the output signal amplitude with the desired image amplitude within the SW is imposed, while free phase is left on the whole DPW. On the right: corresponding hologram phase pattern after 200 iterations. On the hologram plane, direct amplitude-elimination and partial phase-quantization are performed at each step. Evolution of diffraction efficiency *η* (**d**) and signal-to-noise ratio (SNR) (**e**) during algorithm convergence to an optimized design of the computer-generated hologram for illumination with an OAM beam with indices (*p* = 1, *ℓ* = 1) (**a**–**c**).

We chose three different images with increasing complexity for the design of the associated CGHs. The first one exhibits the official Logo of the University of Padua (UniPD logo) and is characterized by having a bitmap format with pure black and white pixels (Fig. 3(a)). The second one shows two intersecting ‘H’ characterized by having grayscale 8 bit/channel (Fig. 3(b)), while the third one represents a wolf portrait with a 8 bit/channel grayscale showing many finer details (Fig. 3(c)). In all cases, the input image is centered in the signal window with a size of 200 × 200 pixels, while the total size of the diffractive optical element is composed of 400 × 400 pixels (Fig. 4(c)).

Starting from the input signal enclosed by a signal window on the image plane, the far-field is brought back to the hologram plane using the inverse Fast Fourier Transform and normalized with respect to the incident illumination, as suggested by eq. (2). The quadratic term in eq. (3) is included in the azimuthal phase of the input field, therefore the image of the computed Fresnel hologram will be at focus on a plane at a distance *z* from the hologram (fixed at 40 cm). Within this iteration procedure, the numerical algorithm generates a continuous complex spectrum in the holographic plane, denoted with *H*_*i*_ in Fig. 3(d). Since the selected lithographic protocol can reproduce phase-only patterns with discretized values of phase, this restriction must be considered and properly implemented in the code. For the benefit of the reader we briefly outline the salient points of the algorithm and we refer to Supplementary Information S2 for more details. A quantization operator *Q* is applied in the hologram plane, within each iteration, performing a direct amplitude-elimination and a direct partial quantization of phase^39^. This operator can be factorized into two parts *Q* = *P·A*, where *P* and *A* are the phase and the amplitude operators, acting on the hologram phase and amplitude respectively. During this step, the hologram transmission function *H*_*i*_ is first normalized by its amplitude, obtaining a phase-only complex spectrum A[*H*_*i*_] = *exp*(*i*Ω), where the phase Ω varies continuously, *modulo* 2π, in the range [0, 2π). Afterwards, by applying the operator *P*, a partial discretization is carried out over a discrete finite set of *M* angles {*γ*_*j*_} (*j* = 1, …, *M*), usually equidistant, being *M* the number of thickness levels of surface-relief hologram pattern to be fabricated. This is performed by dividing the range [0, 2π) into *M* intervals, centered on the values *γ*_*j*_, and substituting at each point (*m, n*) of the hologram the phase Ω_mn_, *modulo* 2π, with the nearest neighbour in the set {*γ*_*j*_}. Such phase quantization is partial, since the half-width of the intervals is not fixed and increases linearly with the iteration number until the whole unitary circle is covered. Therefore, at each iteration, the phase values falling inside the intervals are substituted as described above, otherwise they are left unchanged until the next iteration.

This quantization process is obviously expected to affect the final quality of the image, introducing noise in the reconstruction plane. However, this noise can be reduced by replacing the amplitude within the signal window with the desired amplitude of the original signal, while leaving both phase and amplitude free outside the signal window, where the noise is substantially relegated. Then, the loop is repeated using the output signal as input field for the next iteration step (see Fig. 4).

With the progress of the iterations, the algorithm converges to an optimized design of the holographic pattern. The convergence can be checked in real-time evaluating the signal to noise ratio (SNR) and the diffraction efficiency *η* ^40^. The SNR gives the correlation between input signal and reconstructed signal and is defined as:

8 $$SN{R}_{i}=\frac{\sum _{m,n}{|{h}_{i,mn}|}^{2}}{\sum _{m,n}{|{h}_{i,mn}-{h}_{0,mn}|}^{2}}$$

where *h*_*i,mn*_ is the signal at the pixel (*m*, *n*) at the *i*th step and *h*_*0,mn*_ is the corresponding signal in the reference image. The diffraction efficiency *η* is typically defined as the ratio of the signal energy within the signal window chosen as an active part of the reconstruction plane, and the total energy in the reconstruction (object) plane, given by:

9 $${\eta }_{i}=\frac{\sum _{m,n\in SW}{|{h}_{i,mn}|}^{2}}{\sum _{m,n}{|{h}_{i,mn}|}^{2}}$$

where *W* stands for the set of pixels inside the signal window.

### Fabrication

Phase-only diffractive optical elements are fabricated as surface-relief patterns of pixels. This 3-D structures can be realized by shaping a layer of transparent material, imposing a direct proportionality between the thickness of the material and the local phase delay. Electron beam lithography is the ideal technique to fabricate 3D high resolution profiles^41,42^. By modulating the local dose distribution, a different dissolution rate is induced in the exposed polymer, giving rise to different resist thicknesses after the development process. In this work, the SPP and DOE patterns were written on a PMMA resist layer with a JBX-6300FS JEOL EBL machine, 5 nm resolution, working at 100 KeV with a current of 100 pA. The substrate used for fabrication is glass-coated ITO with low surface resistivity (8–12 Ω) in order to ensure a good discharge of the sample during electron beam lithography. After the exposure, the resist is developed in a temperature-controlled developer bath for 60 s.

At the experimental wavelength of the laser (*λ* = 632.8 nm), PMMA refractive index results *n*_PMMA_ = 1.489 from spectroscopic ellipsometry analysis (J.A. Woollam VASE, 0.3 nm spectral resolution, 0.005° angular resolution). The height *h*_*k*_ of the pixels of the *k*th layer for normal incidence in air is given by

10 $${h}_{k}=\frac{k-1}{M}\frac{\lambda }{{n}_{PMMA}-1}$$

being *M* the total number of phase levels, *k* = 1…, *M*. The fabricated CGHs are 400 × 400 pixels square matrices with *M* = 16 phase levels. Each pixel is 3.125 × 3.125 μm^2^, therefore the total area of each sample is 1.250 × 1.250 mm^2^.

**Figure 5:**
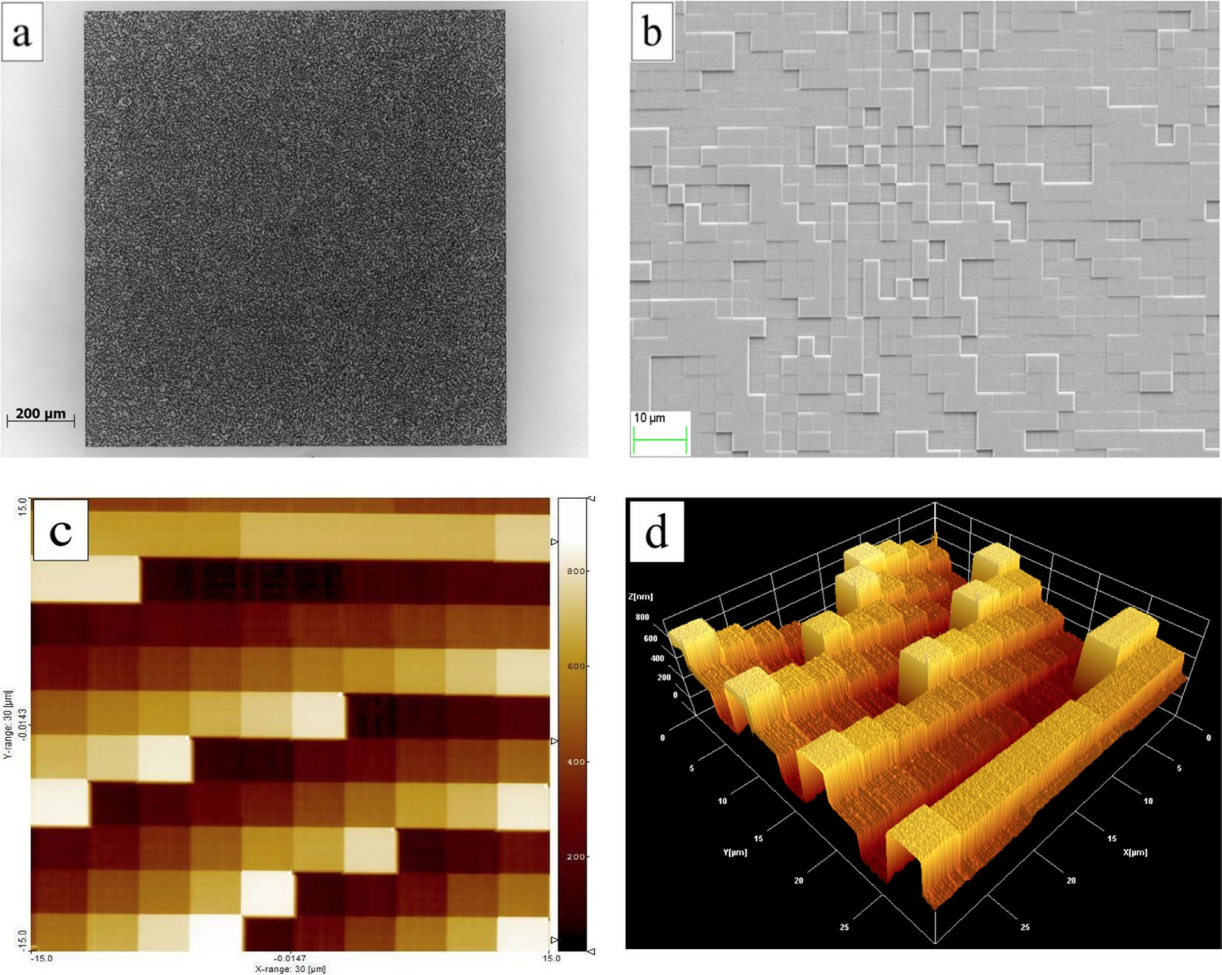
Optical microscopy (**a**), scanning electron microscopy (**b**) and atomic force microscopy (**c**,**d**) of a PMMA phase-only CGH for pixel size: 3.125 × 3.125 μm^2^. CGH total size: 1.250 × 1.250 mm^2^. Working wavelength *λ* = 632.8 nm. 16 phase levels.

Inserting the given laser wavelength and PMMA refractive index in the previous equation, we get: *h*_1_ = 0 nm, *h*_16_ = 1213.2 nm, Δ*h* = 80.9 nm. The quality of the fabricated structures has been assessed using optical microscopy (Fig. 5(a)), Scanning Electron Microscopy (SEM) (Fig. 5(b)) and Atomic Force Microscopy (AFM) (Fig. 5(c,d)). Experimental height values have been compared with the nominal ones exhibiting a remarkable accordance within the experimental errors, estimated considering surface roughness (see Supplementary Figure 1). Spiral phase plates have been fabricated in PMMA on a transparent glass substrate with the same lithographic process, defining the spiral phase ramp with 256 levels^29^. The total thickness, regarding the given wavelength and PMMA refractive index, is 1294.1 nm.

### Optical characterization

**Figure 6:**
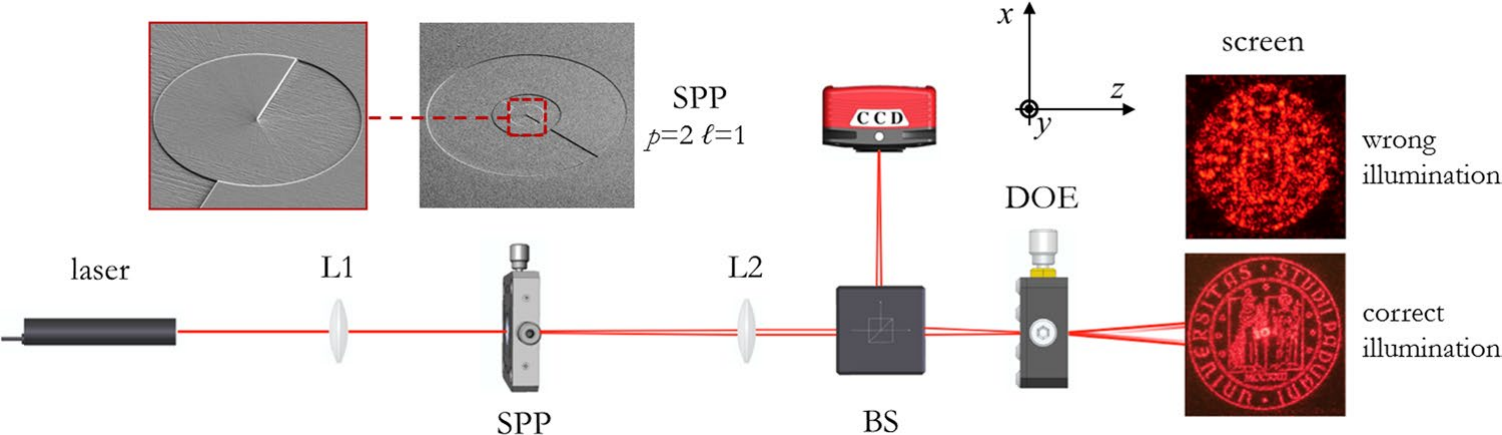
Scheme of the optical characterization setup. Laser source (*λ* = 632.8 nm), first lens (L1), spiral phase plate (SPP), second lens (L2), beam splitter (BS), phase only diffractive optical element (DOE), camera for analysis of the DOE input beam (CCD), screen for revealing the decoded information. Experimental images are reported for a computer-generated hologram encoding UniPD logo, in case of correct (*p* = 1, *ℓ* = +1) and wrong Gaussian illumination. Inset SEM images: details of SPP for the generation of OAM beam with *p* = 2, *ℓ* = +1. The University of Padova logo is © University of Padova and used with permission.

The optical characterization setup was mounted on an optical table (see scheme in Fig. 6). The Gaussian beam was emitted by a HeNe laser source (HNR008R, Thorlabs, *λ* = 632.8 nm, waist *w*_0_ = 240 μm, power 0.8 mW). The polarized beam (LPVISE100-A, Thorlabs) was resized and focused on the selected spiral phase plate. By adjusting the distances from the laser source to the first lens of focal length *f*_1_ = 25 cm and the SPP, the beam-waist is reduced to *w*_1_ = 130 μm. Then the transmitted OAM beam was collimated by a second lens of focal length *f*_2_ = 7.5 cm in *f-f* configuration, and a beam-splitter was used to both collect the intensity profile of the generated OAM beam content and to correctly illuminate the holographic pattern. The field profile was collected with a CCD camera (DCC1545M, Thorlabs, 1280 × 1024 pixels, 5.2 μm pixel size, monochrome, 8-bit depth). Far-field images were collected using Nikon D750 camera.

**Figure 7:**
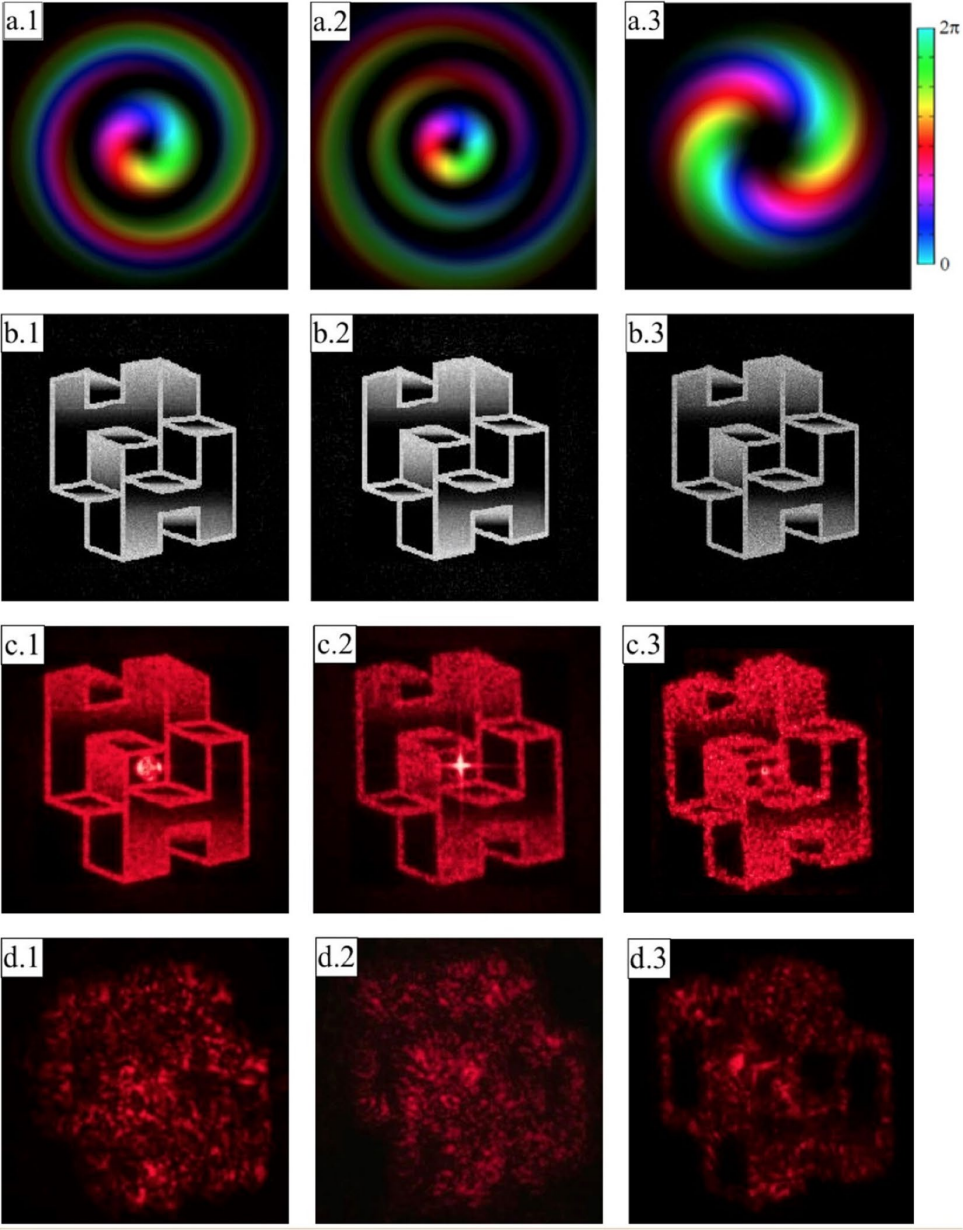
(**a**) Experimental beam generated with SPPs for *p* = 1, *ℓ* = +1 (a.1), *p* = 2, *ℓ* =  +1 (a.2), *p* = 0, *ℓ* =  +2 (a.3). Brightness and colours refer to intensity (experimental) and phase (theoretical) respectively. (**b**) Simulated image for correct illumination with *p* = 1, *ℓ* = +1 (b.1), *p* = 2, *ℓ* =  +1 (b.2), *p* = 0, *ℓ* = +2 (b.3). (**c**) Experimental output in case of correct illumination with *p* = 1, *ℓ* =  + 1 (c.1), *p* = 2, *ℓ* = +1 (c.2), *p* = 0, *ℓ* = +2 (c.3) or wrong Gaussian illumination *p* = 0, *ℓ* = 0 (d.1, d.2, d.3).

The hologram phase pattern for the same image (Fig. 3(b)) has been computed for three different OAM beams: (*p*, *ℓ*) = (1, +1) (Figs. 7(a–d.1)), (*p, ℓ*) = (2, +1) (Fig. 7(a–d.2)), (*p, ℓ*) = (0, +2) (Fig. 7(a–d.3)). As expected, the image which correctly appears under the right illumination (Fig. 7(c.1–3)) is not recognizable with standard illumination (Fig. 7(d.1–3)).

**Figure 8:**
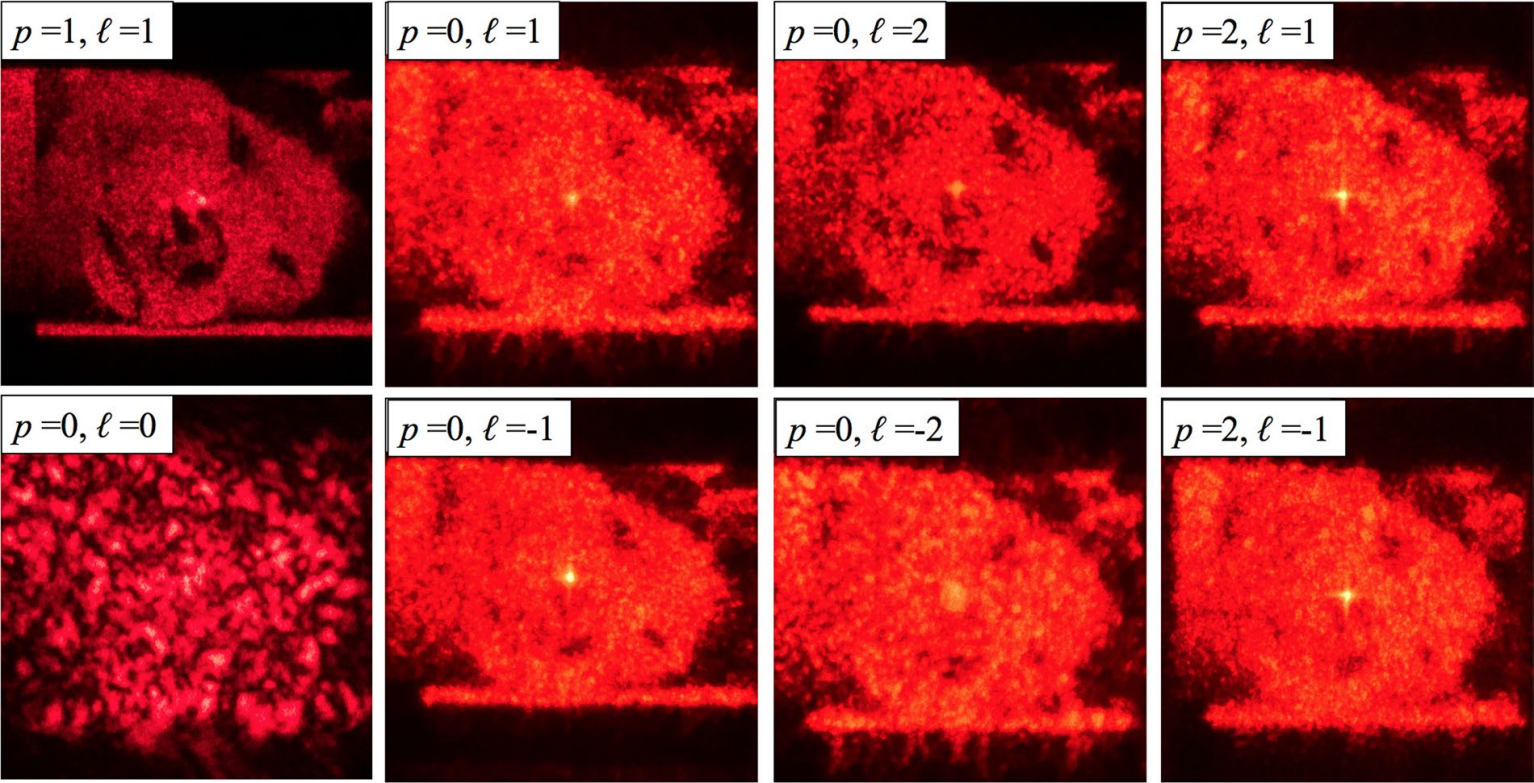
Experimental output of the computer-generated hologram encoding 8 bit/channel grayscale picture, designed for (*p, ℓ*) = (1, +1), under illumination with several spiral phase plates. While the encoded image clearly appears, as expected, when the hologram is illuminated by the correct SPP, under wrong SPP illumination the image details are no longer recognizable.

Then, the fabricated hologram encoding the wolf portrait (Fig. 3(c)), computed for SPP illumination with indices (*p*, *ℓ*) = (1, +1), has been tested with several SPPs shaping the incident illumination, in order to test the optical response of the optical element to input OAM beams different from the optimal one. Again, for wrong SPP illumination, noise increases and the image is no longer clearly visible (Fig. 8).

**Figure 9:**
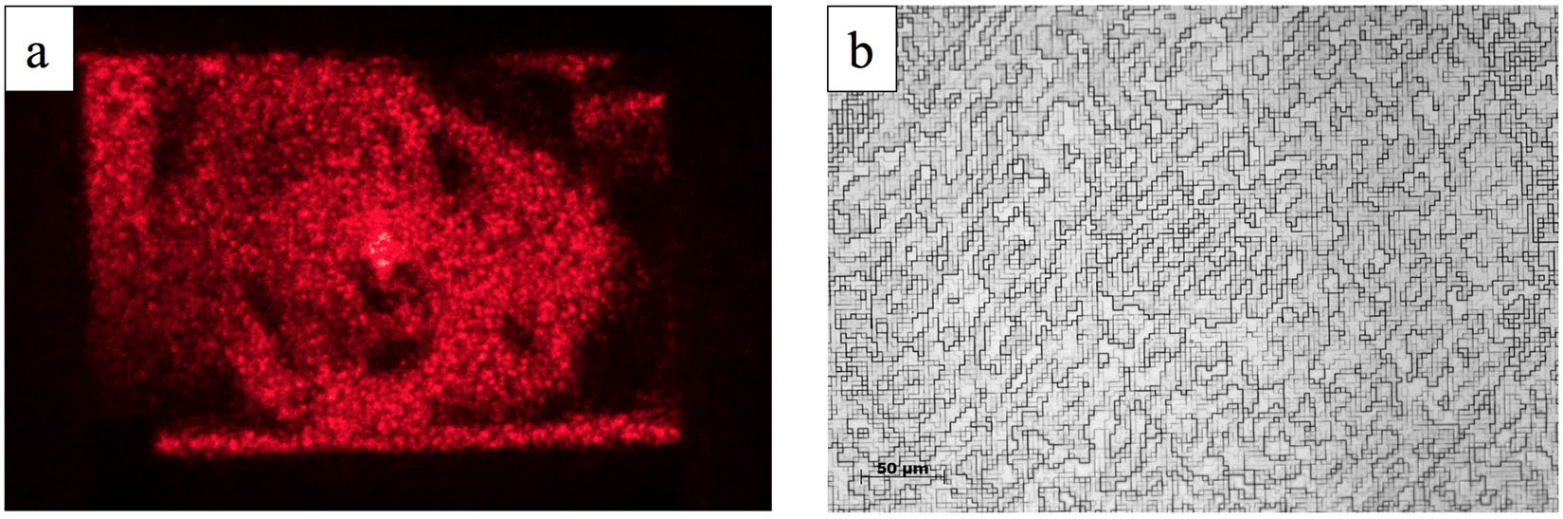
(**a**) Far-field images from holographic-master replica under correct OAM illumination (*p* = 1, *ℓ* = +1), and (**b**) optical microscope inspection.

A soft-lithography replica of a holographic PMMA master has been optimized in order to set-up an easy, fast and low-cost fabrication procedure. The optical and morphological characterizations of the generated copies demonstrate exceptional reliability in replicating the hologram 3D structures (see Fig. 9 and supplementary information Figure S3).

**Figure 10:**
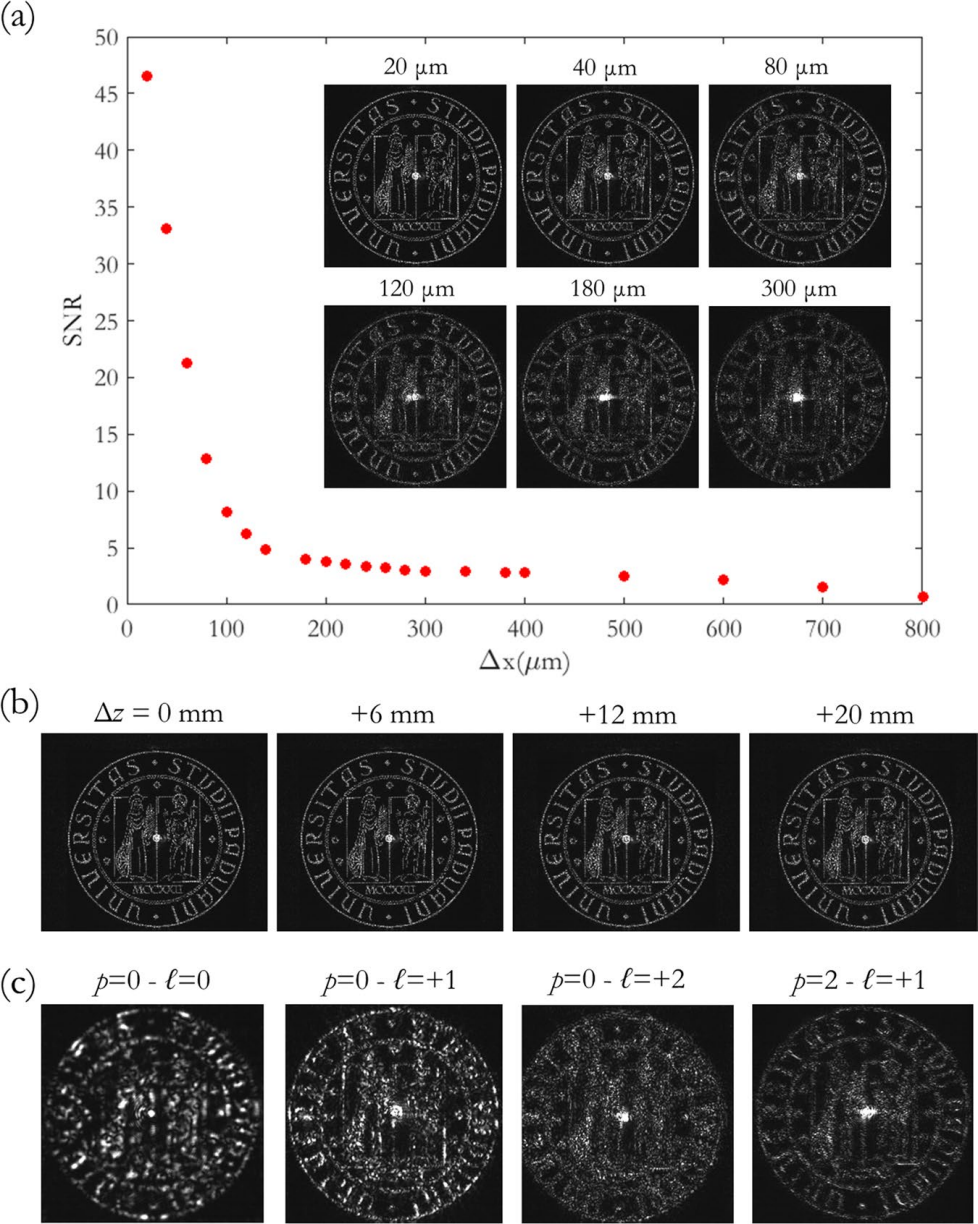
(**a**) Analysis of the UniPD logo CGH for increasing lateral misplacement with respect to the input beam with indices (*p* = 1, *ℓ* =  +1). Experimental SNR (calculated with respect to the aligned configuration) as a function of the lateral shift Δ*x*. Inset pictures: experimental far-field for several values of Δ*x*. (**b**) Experimental far-field for increasing positions of the CGH along the *z*-axis, i.e. along the beam axis. (**c**) The same CGH, centered and aligned with respect to the input beam, illuminated with OAM beams carrying index values different from the original ones. The University of Padova logo is © University of Padova and used with permission.

Since the CGH phase patterns are computed to be illuminated by a specific intensity and phase spatial distribution, the image reconstruction is expected to be sensitive to misplacements of the decoding beam with respect to the sample position. In order to analyse the far-field image quality as a function of the sample displacement, we selected the computer-generated hologram encoding the UniPD logo decoded for OAM illumination with indices (*p*, *ℓ*) = (1, +1) and we collected the far-field image under the correct beam indices and size but for increasing radial and axial shifts of the beam. Specifically, since the input beam is axially symmetric, we moved the hologram along the *x*-axis positive direction, on a plane perpendicular to the beam axis and located on the beam waist. As Fig. 10(a) shows, the reconstructed image is gradually destroyed for increasing lateral displacement and the details are no longer clearly distinguishable for shift values beyond 90 μm, when the SNR, calculated with respect to the aligned case, drops to values below 10. This threshold value corresponds to 33% of the beam waist radius (*w*_0_ = 0.275 mm) of the decoding beam and about 14% of the hologram half-size (0.625 mm). For greater shifts, the zero-order term becomes dominant and the finest details of the image are not recognizable. However, it is worth noting that this effect is less remarkable than in the case of wrong input illumination, i.e. with wrong radial and azimuthal indices.

The alignment along the beam axis is less critical, as shown in Fig. 10(b), where several far-field images are reported for increasing shift in the *z* direction up to 2 cm, which corresponds to 26.7% of the focal length *f*_2_ used for OAM-beam focusing on the sample (*f*_2_ = 7.5 cm). This tolerance in the hologram position is referable to the amount of the Rayleigh range for the considered beam, which is around 18.8 cm, one order of magnitude greater than the considered *z*-misplacement. A shift of 2 cm in the z-direction corresponds to an increase in the beam radius around 0.5%, therefore the intensity distribution is roughly the same. However, the incident wavefront is not planar, since the sample is not placed at the beam waist, therefore the image is expected to be focused at a slightly shifted position.

In supplementary information section S3 additional figures are reported regarding the previous analysis. In addition, in order to further enhance the possibility of future applications in Track and Trace using these diffractive optical elements, an image representing a QR-code has been computed and analyzed under OAM illumination (see supplementary information Figure S7) and its tolerance to lateral shifts of the incident beam was checked.

## Discussion

In this work, we present the first attempt of a complete design and fabrication procedure of computer-generated holograms encoding information for illumination with structured light beams carrying specific distributions of intensity and phase. In particular, we consider beams carrying orbital angular momentum, generated with high-order spiral phase plates enabling both the transfer of topological charge to the input beam and the generation of a multi-ring intensity pattern. An iterative Fourier transform algorithm has been implemented for the computation of an optimized phase pattern for the selected input image and incident field. The computation of the hologram pattern for the given input beam imposes a one-to-one correspondence between the generated hologram and the SPP and therefore increases the security level of these diffractive optics, so that the encoded information cannot be addressed without the correct illumination key. This result has been demonstrated with the design and optical test of several samples, fabricated with high-resolution electron-beam lithography. The optical characterization demonstrates that the encoded image appears, as expected, only when the hologram is illuminated with the correct input illumination, otherwise the information is not recognizable, neither with standard Gaussian illumination nor different OAM beams.

Considering security applications, someone could in principle attempt to brutally-force the information encoded in the hologram by sequentially generating a varying input illumination by spanning all the values of the indices (*p*, *ℓ*) over an arbitrary set until the correct values are guessed, for instance by using a computer-controlled spatial light modulator. A hacking attempt of this kind can be prevented or at least dissuaded by the following considerations. Firstly, a crucial role is represented by the geometrical size of the beam. As theoretically demonstrated in^43^, the indices *p* and *ℓ* define an orthonormal set of modes only for a specific value of the beam-waist parameter, therefore beams with the same *ℓ* and different *p* are no more orthogonal if they differ in the beam size as well. As numerically shown in^10^, for a given set of indices, the image of the encoded hologram clearly forms only in the neighbourhood of the optimal beam-waist. Differently to *p* and *ℓ*, which can assume only discrete integer values, the beam-waist is a continuous parameter and the dimensionality of the decoding optical key is therefore remarkably increased. Secondly, the complexity of the decoding optical key can be improved by considering a superposition of two or more OAM-beams with different values of *ℓ* and *p*, generating more complex distributions of amplitude and phase^44^, instead of a symmetric one with integer OAM as presented in this work. Therefore a further parameters set would be represented by the different weights of the several modes constituting the input beam, which are continuous parameters assuming in principle any values in the range (0, 1).

Electron-beam lithography provides a high-resolution lithographic technique, allowing the fabrication of concealed security holograms with a resolution far higher than common dot-matrix optical devices^45,46^, usually fabricated with interferential-lithography systems. One may consider the cost of the EBL as a drawback, but in fact it is the cost of the increased quality and resolution of the holograms. Moreover, we have demonstrated the replica process of one high-quality hologram to produce several identical copies by soft lithography methods, which are well-considered as a super-economical technique.

In supplementary information S5, the replica process of a holographic PMMA master is explained in details. Soft lithography technique provides an exceptional reliability in reproducing the exact 3D structures in an easy, fast and low cost process.

The experimental optical bench exploited in this work for the optical characterization of the fabricated diffractive optics, is clearly cumbersome and unhandy in the view of commercial and industrial applications. However, further improvements and integration can be performed. In particular, the SPP can be embodied to the laser source exploited for hologram inspection. The SPP phase pattern will be properly optimized for the size of the beam exiting the laser, and a lens term can be integrated in the SPP phase profile in order to focus the OAM beam at a proper distance, without the need of a further lens as shown in Fig. 6. Misalignment analyses showed a good tolerance in the hologram positioning with respect to the beam focal plane, provided misplacement is one order of magnitude lower than the Rayleigh range of the beam. On the other hand, the alignment of the hologram with respect to the OAM beam singularity is much more critical. A threshold misalignment around 30% of the beam waist radius has been shown in case of OAM beam with indices (*p*, *ℓ*) = (1, +1), corresponding to about 90 μm in the considered optical setup. However, this value is far greater than the accuracy of mechanical alignment systems, therefore such security optical elements could be detected and decoded by electronically-controlled readers which control the position of the input beam with a micrometric precision. A by hand inspection would require an increase of this tolerance value, for instance increasing the hologram size and the waist of the decoding illumination.

By engineering this new type of diffractive optical elements, which correctly decode visual information only when illuminated with light owning a specific spatial distribution of intensity and phase, we expand the available range of security optical devices and of possible applications of structured light, whenever information needs storing with increased security and counterfeit prevention. The miniaturized size of the fabricated optics allows them to be either integrated or concealed onto greater-size optical elements, such as 2D/3D holograms and other types of overt security devices to be applied on documents and products.

## Methods

### Numerical simulations

A custom MATLAB code based on IFTA was implemented in order to compute the phase-only diffractive optical element specifically designed for OAM illumination (see Supplementary Information S2 for details about the algorithm).

### Electron beam lithography

All 3D multilevel structures have been fabricated in a 2 µm thick PMMA resist with a molecular weight of 950 k (kg/mol), spin-coated on a 1.1 mm thick ITO coated soda lime float glass substrate and pre-baked for 10 min at 180 °C on a hot plate. For the grey-scale lithography step, a dose-depth correlation (contrast curve) was used. Contact profilometry was performed to determine the remaining resist heights. Dose-to-clear value (complete removal of PMMA) was found to be 566 µC/cm^2^. CGH patterns were written with a JBX-6300FS JEOL EBL machine, 12 MHz, 5 nm resolution, working at 100 KeV with a current of 100 pA. The presence of the ITO layer was necessary in order to ensure a good discharge of the sample during electron beam lithography. A dose correction for the compensation of proximity effects has been applied. This compensation is required both to match layout depth with the fabricated relief and to obtain a good shape definition, especially in correspondence of the phase steps. Exposed samples were developed under slight agitation in a temperature-controlled developer bath for 60 s. Deionized water: isopropyl alcohol (IPA) 3:7 was found to be the most suitable developer, giving optimized sensitivity and contrast characteristics as well as a minimized pattern surface roughness at 20 °C. After development, the samples were gently rinsed in deionized water and blow-dried using nitrogen flux. Different techniques have been used in order to assess sample quality: tapping-mode atomic force microscopy, optical microscopy and scanning electron microscopy.

### Soft-lithography replica

A suitable amount ofthe elastomer Sylgard 184 polydimethylsiloxane (PDMS)base is mixed with the catalyst in a weight ratio of 10:1 respectively, stirred for a while, and then cast onto the surface of the EBL fabricated master. During the PDMS pouring and mixing, creation of bubbles is inevitable, so the container is placed in a desiccator for30–45 minutes to de-gas it and remove the trapped air bubbles. The sunken master with PDMS prepolymer is then placed in an oven at the temperature of 100 °C to be cured for 35 minutes, then was left in a freezer for 5 to 10 minutes to cool down. This shrinks the PDMS slightly and helps when peeling the samples out of their molds.

To fabricate the replica of the mold, a UV-curable photopolymer (Norland Optical Adhesive 74) is dropped onto a glass substrate. The PDMS mold is overlaid on it (with the patterned side facing the liquid) and is pushed a bit to form the contact. Finally, the photopolymer is cured under UV light for 30 seconds, after which the PDMS mold is carefully peeled off to get the final replica.

### Optical characterization

The characterization setup was designed and assembled on an optical table with gimbal piston isolators (refer to Fig. 6). The Gaussian beam was emitted by a HeNe laser source (HNR008R, Thorlabs, *λ* = 632.8 nm, waist *w*_0_ = 240 μm, power 0.8 mW). The polarized beam (LPVISE100-A, Thorlabs) impinges on the corresponding spiral phase plates, mounted on a sample holder with micrometric drives (ST1XY-S/M, Thorlabs, travel 2.5 mm, resolution 10 μm). By adjusting the distances from the laser source to the first lens of focal length *f*_1_ = 25 cm and the SPP, the beam-waist was reduced to *w*_1_ = 130 μm. Then the transmitted beam was collimated by a second lens of focal length *f*_2_ = 7.5 cm and a 50:50 beam-splitter was used to both collect the intensity profile of the generated OAM beam content and to correctly illuminate the holographic pattern. The field profile was collected with a CCD camera (DCC1545M, Thorlabs, 1280 × 1024 pixels, 5.2 μm pixel size, monochrome, 8-bit depth). The holographic sample was fixed on a vertical *XY* translation mount with micrometric drives (ST1XY-S/M, Thorlabs, travel 2.5 mm, resolution 10 μm), and the far-field was projected on a white screen. The projected far-field images were collected using Nikon D750 camera. During misalignment analyses, the far-field was recorded using a second DCC1545M (Thorlabs) CCD camera. The hologram was moved with respect to the beam axis by rotating the ST1XY-S/M micrometric screw, providing a shift of 10 μm per graduation. For *z*-misalignment analysis, both hologram holder and CCD camera were mounted on an optical rail (RLA150/M, Thorlabs) with mm graduation.

## Electronic supplementary material

Supplementary information
