# Supplementary material for: Design, fabrication and characterization of Computer Generated Holograms for anti-counterfeiting applications using OAM beams as light decoders
Source: Sci Rep. 2017 Dec 21;7:18011. doi: 10.1038/s41598-017-18147-7 (PMC5740128; doi:10.1038/s41598-017-18147-7)
Supplement: Supplementary file 1 — Supplementary information [file 41598_2017_18147_MOESM1_ESM.pdf]

Supplementary information

Design, Fabrication and characterization of Computer Generated Holograms for anti-counterfeiting applications using OAM beams as light decoders

Gianluca Ruffato[1,2], Roberto Rossi [1,2], Michele Massari[1,2], Erfan Mafakheri[1,2], Pietro Capaldo[2, 3] and Filippo Romanato[1,2,3]*

[1]Department of Physics and Astronomy 'G. Galilei', University of Padova, via Marzolo 8, 35131 Padova, Italy

[2]LaNN, Laboratory for Nanofabrication of Nanodevices, EcamRicert, Corso Stati Uniti 4, 35127 Padova, Italy.

[3]CNR-INFM TASC IOM National Laboratory, S.S. 14 Km 163.5, 34012 Basovizza, Trieste, Italy

[*]Corresponding author: filippo.romanato@unipd.it

S1. AFM ANALYSIS

Atomic Force Atomic (AFM) microscopy has been performed in tapping-mode configuration for a 16-level phase-only computer-generated hologram (CGH) in PMMA, working in transmission at λ=632.8 nm. Experimental depth values are: d_0=0 nm, d_1=82.7 nm, d_2=161.2 nm, d_3=248.8 nm, d_4=325.9 nm, d_5=401.0 nm, d_6=494.0 nm, d_7=571.0 nm, d_8=636.0 nm, d_9=721.0 nm, d_{10}=810.0 nm, d_{11}=885.0 nm, d_{12}=973.0 nm, d_{13}=1055.0, d_{14}=1138.0 nm, d_{15}=1219.0 nm, d_{16}=1291.0 nm. In Supplementary figure 1, a 3D AFM reconstruction is reported for a small area of 30 x 30 μm^2.

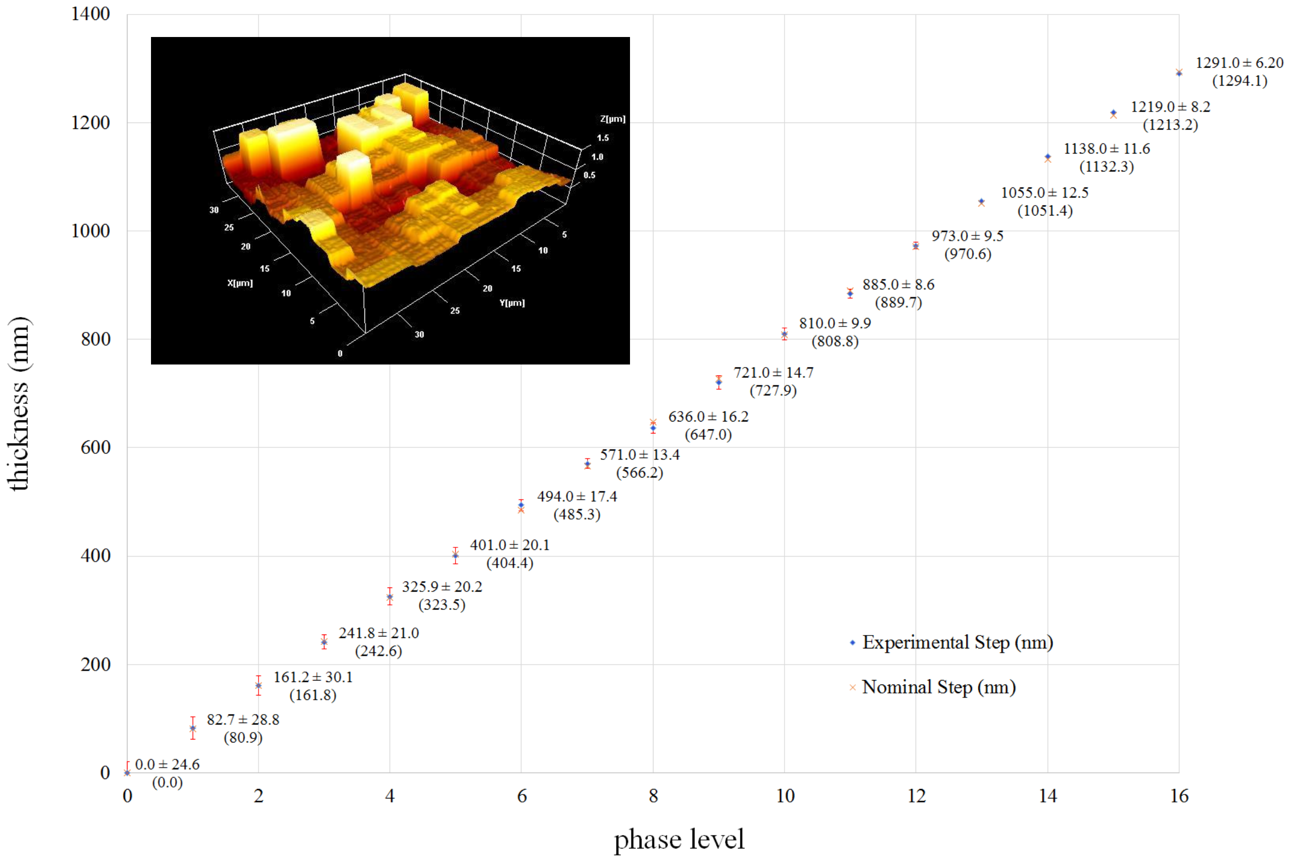

Supplementary Figure 1. Analysis of 30 x 30 μm^2 (spanning about 10 x 10 pixels) zone of the 16-level holographic DOE. Comparison between experimental (rhombus with error bars) and nominal (cross) heights is reported.

Experimental values are compared with the nominal ones exhibiting a remarkable accordance within the experimental errors, estimated by considering surface roughness. Roughness root-mean-square (RMS) increases from 6 nm to 25 nm, for the lowest and highest depth level respectively.

S2. ITERATIVE FOURIER TRANSFORM ALGORITHM (IFTA)

The main task of the iterative algorithm is to transform the complex hologram spectrum function into a discrete form (to a binary or 2^n-level phase-only form). This discretization is done sequentially during the process. Obviously, such quantization introduces noise in the reconstruction plane, which is removed, or at least significantly reduced, by iteratively replacing the amplitude of a newly calculated noisy signal, within the signal window, with the desired amplitude of the original signal. This modified signal is used as input for the following step. The quantization represents a transformation of the hologram function to the desired form while maintaining reconstructed image quality within defined constraints. In mathematical terms, the whole IFTA procedure can be resumed as it follows:

1. h_i is the signal at the input of the *i*th iteration loop in the object plane. Before normalizing with respect to the incident field U^i, comprehensive of the Fresnel term, the inverse fast Fourier transform allows passing to the hologram plane where the hologram function H_i is defined as:

$$H_i = FFT^{-1}\left[h_i\right]/U^i \tag{1}$$

2. The quantization operator Q is applied in the hologram space and the phase function H_i' is generated:

$$H_i' = Q\left[H_i\right] \tag{2}$$

When iteratively shaping multilevel profiles within the IFTA method, there remains an open questions of how to eliminate iteratively the amplitude of the spectrum and how to correctly perform phase quantization. The operator Q should, in principle, consist of two parts:

$$Q = P \cdot A \tag{3}$$

where P and A are respectively the phase and the amplitude operators, which performs the quantization at each iteration of the hologram phase and amplitude respectively. As far as the

amplitude operator is concerned, the *direct (single-step) amplitude elimination* is usually applied, with very good results, that is:

$$A[x] = \frac{x}{|x|} \tag{4}$$

A correct choice of P operator is crucial for convergence of the algorithm and optimization of the hologram. Phase quantization is performed by iterative expansion of local surroundings of quantization levels, and is represented formally by the operator P. This operator has the form:

$$P[x] = \begin{cases} q_j & x \in \varepsilon_j(i) \\ x & otherwise \end{cases} \tag{5}$$

where $\{q_j\}$ is the set of M target quantization points lying on the unitary circle in the complex plane and $\{\varepsilon_j(i)\}$ is the set of particular surroundings within the ith iteration cycle that simultaneously defines the areas where the replacement of the values takes place. Each surrounding is defined as:

$$\varepsilon_j(i) = \left\{ q \mid \left| angle(q) - angle(q_j) \right| \le e_j(i)/2 \right\} \tag{6}$$

where $e_j(i)$ is the amplitude of the surrounding $\varepsilon_j(i)$, centered on the quantization phase level $angle(q_j)=atan(Im(q_j)/Re(q_j))$, at the ith step. The idea is, at each iteration, to leave a certain number of unprocessed values outside regions adjacent to the quantization levels. In order to make further optimization feasible, it is necessary to keep a sufficient number of these unprocessed values during the process and enable values already processed to jump from one target phase level to either another phase level or to the regions of unprocessed values. On the one hand the quantization ranges $e_j(i)$ can be kept fixed throughout the iteration, equal to the whole spectral range of interest for the given set of quantization levels. In this case a full quantization only would be periodically repeated. On the other hand, $e_j(i)$ can be enlarged gradually step by step, while each actual size of the surroundings is applied only once, that is:

$$e_j(i) = \frac{2\pi}{M}\frac{i}{N} \tag{7}$$

This procedure is known as *one-step approach with partial phase quantization*. Alternatively, this procedure can be iterated, in the form of the two-step approach or repeated whole partial quantization. A further scheme consists in the repetition of each partial quantization step: two-step approach with repetition of each partially quantizing step. In this last case all expanded surroundings are themselves repeated while the whole quantization is performed once. In this work we applied direct amplitude elimination and one-step partial phase quantization.

3. The simulated reconstruction in the object domain h_i' is given by the fast Fourier transform, once the hologram has been multiplied by the incident field:

$$h_i' = FFT\left[H_i' \cdot U^i\right] \tag{8}$$

4. Finally the new signal h_{i+1} is obtained by the proper replacement of the output signal h_i' with the desired signal within the object window, through the operator R:

$$h_{i+1} = R[h_i'] \tag{9}$$

At this step, the aim is to disturb the partially quantized structure in the smallest possible way. This signal modification, in fact, partially destroys the quantized character of the structure already formed. This detrimental effect should be minimized using a scale factor γ:

$$\gamma_i = \frac{\sum_{m,n} FFT\left[w \cdot h_i'\right] FFT^{\dagger}\left[w \cdot h_0 \cdot \frac{h_i'}{|h_i'|}\right]}{\sum_{m,n\in W} |h_0|^2} \tag{10}$$

where w=1 inside the signal window, otherwise it is null.

5. The current loop is completed, and the iteration goes on until the total number N of iteration is reached

S3: ROBUSTENESS OF THE HOLOGRAM WITH RESPECT TO BEAM MISALIGNMENT

The hologram encoding the UniPD logo decoded for OAM illumination with indices (p, ℓ)=(1, +1) was selected in order to test the robustness of the image reconstruction as a function of the hologram misalignment with respect to the decoding beam. Here additional experimental images are reported, concerning the completion of the analysis described in the main manuscript. In Supplementary Figure 2, the experimental far-field is shown for an increase in the lateral shift of the hologram. As reported in the article, the image is gradually destroyed and many details are no longer clearly distinguishable for shift values greater than 80 μm, where the SNR, calculated with respect to the aligned case, drops to values below 10. This threshold value corresponds to 30% of the beam waist radius (w_0=0.275 mm) of the decoding beam and about 13% of the hologram half-size (0.625 mm). For greater shift values, the zero-order term becomes dominant and the finest details of the image are no more recognizable.

The alignment along the beam axis is less critical, as shown in Supplementary Figure 3, where several far-field images are reported for a shift of the hologram in the range from -23 to +23 mm with respect to the beam waist plane. This tolerance in the hologram position is ascribable to the value of the Rayleigh range for the considered beam, which is around 18.8 cm, one order of magnitude greater than the considered z-misplacement. A shift of 2.3 cm in the z-direction, corresponds to an increase in the beam radius of around 0.7%.

It is worth noting that the effect of hologram misalignment is less remarkable than in the case of wrong input illumination, i.e. with wrong radial and azimuthal indices. As shown in Supplementary Figure 4, the UniPD logo details are not recognizable if the sample is illuminated with OAM beams different from the correct one, even when the hologram is perfectly aligned and centered on the beam waist plane.

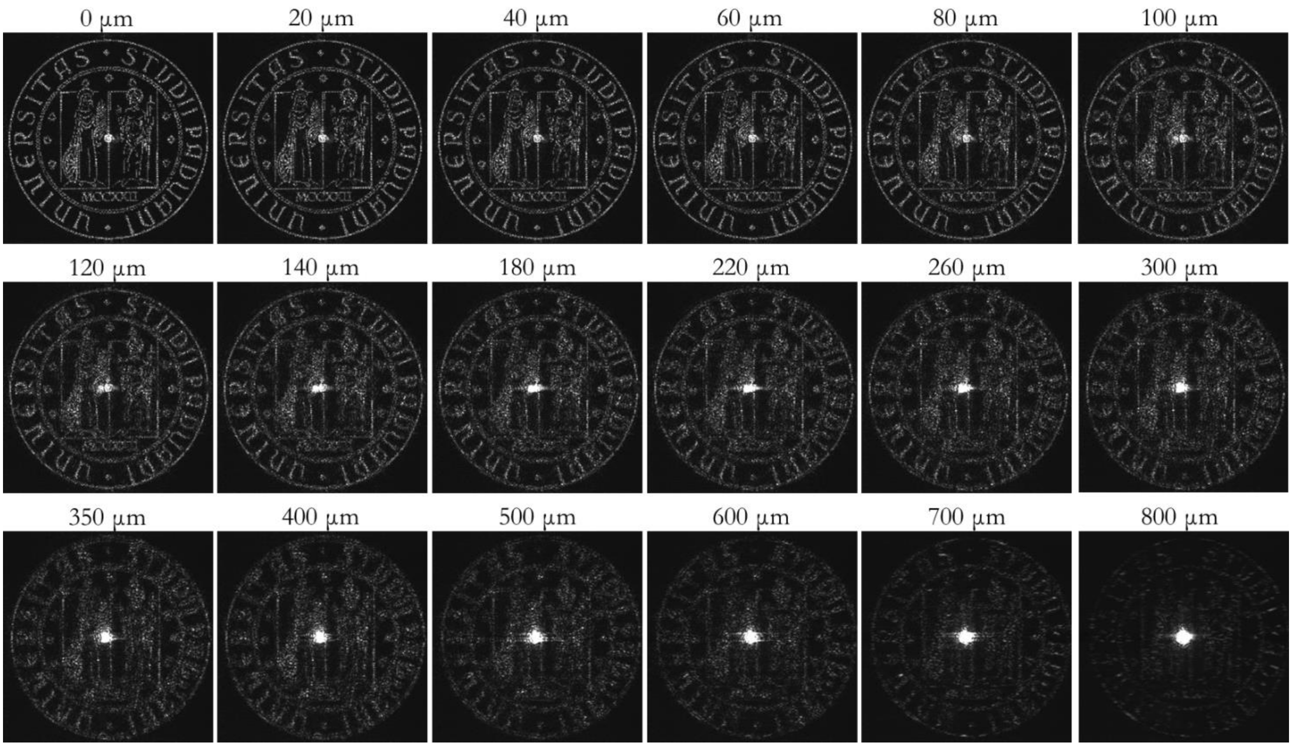

Supplementary Figure 2. Analysis of the UniPD logo hologram for increasing lateral displacement with respect to the decoding input beam with indices (p=1, ℓ=+1). The University of Padova logo is © University of Padova and used with permission.

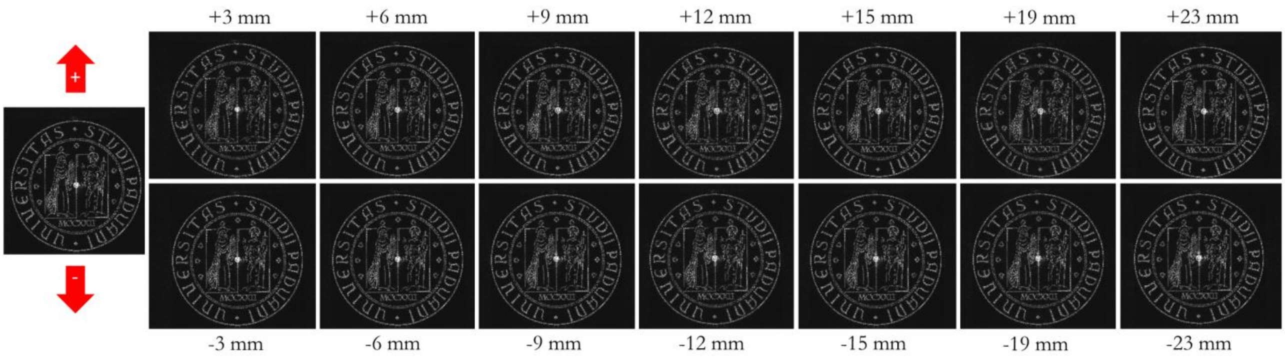

Supplementary Figure 3. Experimental far-field for several positions of the hologram encoding UniPD logo along the *z*-axis, i.e. the beam axis. The reconstructed image exhibits a good reconstruction quality in the considered range. The University of Padova logo is © University of Padova and used with permission.

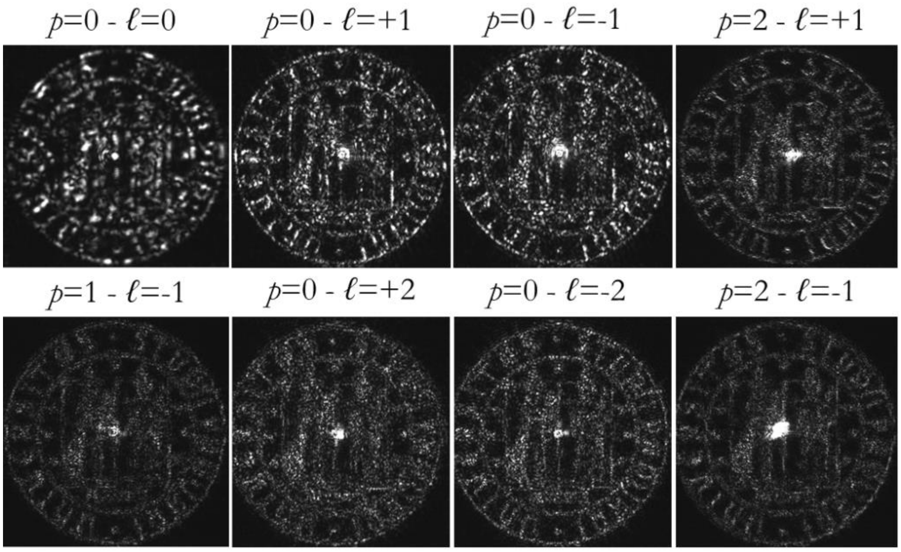

Supplementary Figure 4. Experimental reconstructed image of the hologram in Supplementary figure 2, encoding UniPD logo, centered and aligned to the input beam, illuminated for several OAM beams with wrong indices. The University of Padova logo is © University of Padova and used with permission.

S4. OPTICAL CHARACTERIZATION OF A QR-CODE AS HOLOGRAPHIC IMAGE.

In order to further enhance the range of applications in anti-counterfeiting and brand protection, a holographic QR-code has been used as image for illumination with indices p=1, ℓ=+1 and characterized on the optical bench (refer to Figure 6 for the optical setup).

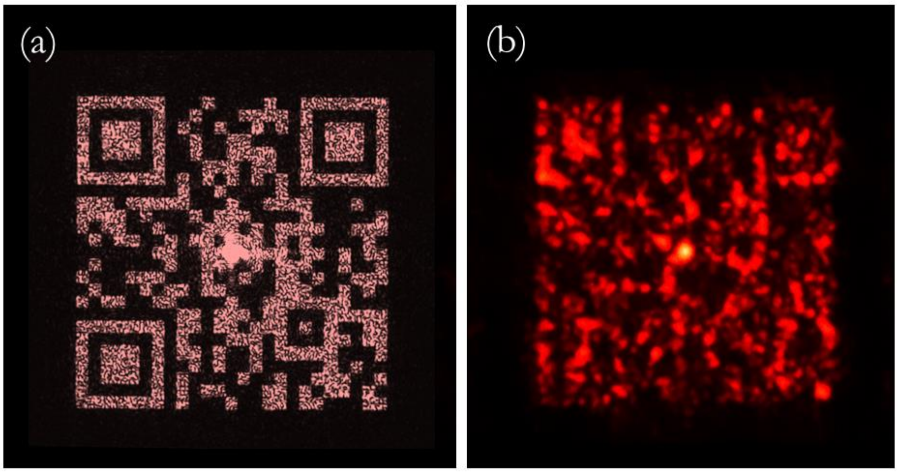

Supplementary Figure 5. Experimental output of a QR-code holographic image. Case of (a) correct OAM illumination for (p=1, ℓ=+1) or wrong (b) Gaussian illumination (p=0, ℓ=0).

When the image is correctly illuminated, it allows the recognition of the http:// address using common mobile APPs such as (QR code reader, Android App). In the presented case, the associated address corresponds to *http://www.lann.it.* Moreover, we considered the quality of the reconstructed image as a function of the input-beam lateral misalignment. As expected, noise increases with lateral shift, and the code is no longer recognizable by QR-reader APPs after a shift around 80-90 μm, which is around the 13%-14% of the hologram half-size and 30% of the input beam waist radius.

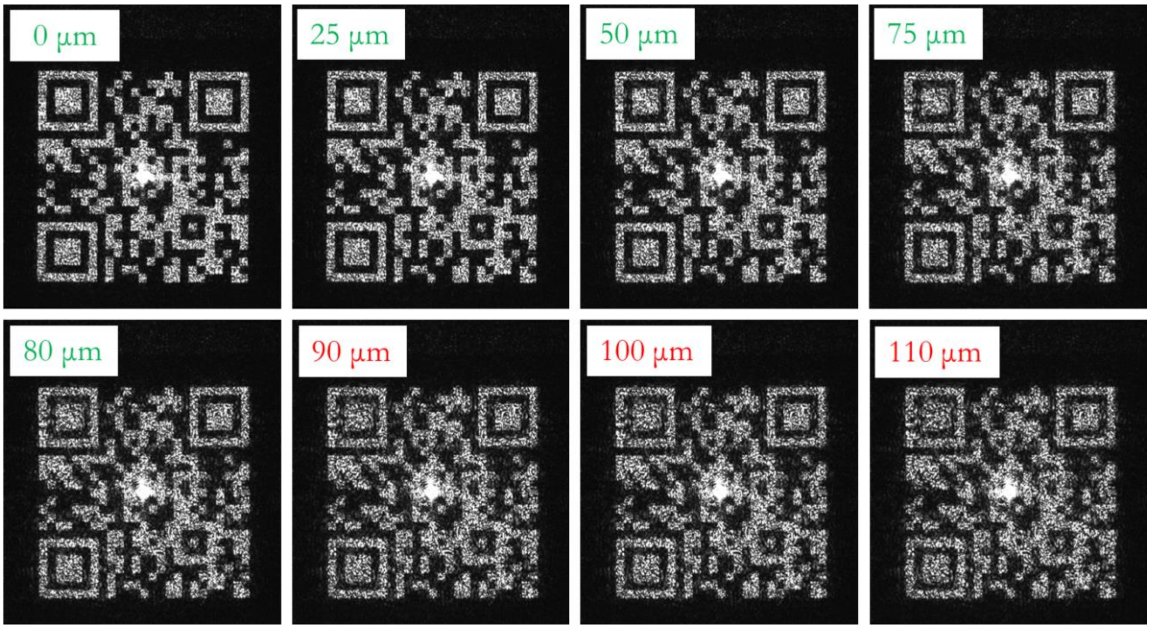

Supplementary Figure 6. Experimental output of a QR-code holographic image encoding the *url* address *http://www.lann.it* for different lateral shifts with respect to the input beam axis. After a displacement around 80 μm the reconstructed image results no longer readable by common QR-reader APPs.

S5. REPLICA WITH SOFT LITHOGRAPHY OF HOLOGRAPHIC DOEs

Fabrication techniques like photolithography, electron-beam lithography, etc. are extremely successful in patterning structures of radiation-sensitive materials (e.g. photoresists or electron-beam resists) on glass or semiconductor surfaces. However significant challenges exist in adapting these lithographic methods for emerging applications and areas of research that require unusual

systems and materials (e.g. those in biotechnology, plastic electronics, etc.), large patterned areas or nonplanar surfaces. These established techniques also involve high capital and operational costs. As a result, older and conceptually simpler forms of lithography – embossing, molding, stamping, writing, etc. – are being re-examined for their potential to serve as the basis for nanofabrication techniques that avoid these limitations. These are altogether known as 'soft lithography', named for their use of soft, elastomeric elements in pattern formation.

The soft lithography process can be arranged in two steps: fabrication of the elastomeric elements and use of these elements to pattern features in geometries defined by the element's relief structure. The original patterned structure from which the stamp is derived is known as the 'master'. Many elastomeric elements can be generated from a single master, and each element can be used several times to replicate the initial master. Elastomeric elements are generated by casting a light- or heat-curable prepolymer against the master. With optimized materials and chemistries, this fabrication sequence has remarkably high fidelity. In fact, recent works shows that relief with nanometric depths and widths can be reproduced accurately.

In this work, a soft lithography process has been finely tuned in order to realize high-fidelity replicas of computer-generated holograms fabricated by high-resolution electron beam lithography. Particular attention has been paid to the optimization of the curing processes in order to best preserve the steepness of the vertical profiles of the original patterns. In addition, materials have been carefully selected in order to ensure the same optical behaviour of the original masters. The quality of the replicas has been tested with optical and AFM microscopy and characterized with an optical setup mounted on an optical table. The optical behaviour of the fabricated replicas is comparable to that of the original CGH masters. The successful transfer of this technique into manufacturing settings for important applications that cannot be addressed effectively with other lithographic methods offers the promising possibility to realize the holographic optical elements

through high-throughput and low-cost realization processes. In Supplementary Figure 7 a scheme of the replica process exploited in this work is reported.

Fabrication of the elastomeric element (*PDMS mold*)*:* a suitable amount of the elastomer Sylgard 184 polydimethylsiloxane (PDMS) base is mixed with the catalyst in a weight ratio of 10:1 respectively, stirred for a while, and then cast on the surface of the EBL fabricated master (PMMA mold). During the PDMS pouring and mixing, creation of bubbles is inevitable, so the container is placed in a desiccator for 30-45 minutes to de-gas it and remove the trapped air bubbles. The sunken master with PDMS prepolymer is then placed in an oven at the temperature of 100°C to be cured for 35 minutes, then left in a freezer for 5 to 10 minutes to cool down. This shrinks the PDMS slightly and helps when peeling the samples out of their molds.

Replica process (*NOA cast*)*:* to fabricate the replica of the mold, a UV-curable photopolymer (Norland Optical Adhesive 74) is dropped on a glass substrate. The PDMS mold is overlaid on it (with the patterned side facing the liquid) and is pushed a bit to form the contact. Finally, the photopolymer is cured under UV light for 30 seconds, after which the PDMS mold is carefully peeled off to get the final replica.

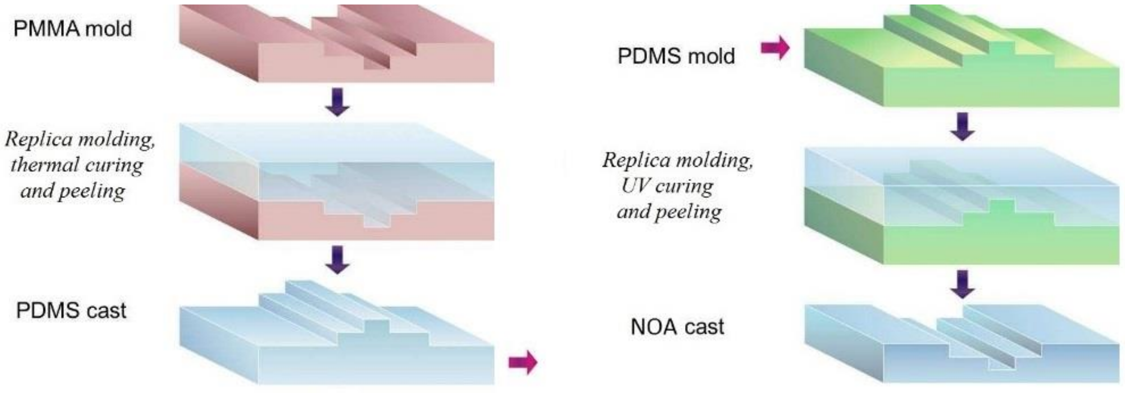

Supplementary Figure 7. Essential scheme of the soft lithography process. On the left: PDMS replica of the master. PDMS is cast on the surface of the master, thermal cured and peeled off. On the right: NOA replica of the PDMS mold. The NOA photopolymer is dropped onto the PDMS mold, UV-light cured and peeled off the mold to get the final cast.
